# Supplementary material for: Host and fungal factors both contribute to cryptococcosis-associated hyperammonemia (cryptammonia)
Source: Microbiol Spectr. 2024 Jun 6;12(7):e03902-23. doi: 10.1128/spectrum.03902-23 (PMC11218482; doi:10.1128/spectrum.03902-23)

Supplemental Material

Table S1: Host factors and cryptococcal culture source, time and frequency from

three patients included in Cryptammonia Study.

| Patient | Isolates | Culture Source | Day of admission culture was obtained | Renal Failure | Liver Failure | NH3 Level closest to time of culture collection within 24 hours. (Normal 30 -53µmol/L ) | Peak or Trough NH3 level within 48 hours after culture obtained* (Normal 30 -53µmol/L) | Time Interval between NH3 levels^#^ (Hours) | Rate of change per hour of plasma NH3 level (µmol/hour) |
| --- | --- | --- | --- | --- | --- | --- | --- | --- | --- |
| A | A1 | Blood Culture  Bacterial/Yeast | Day 1 | Yes | No | 205 | 692 | 36.8 | 13.23 |
| A | A2 | Blood Culture Bacterial/Yeast | Day 3 | Yes | No | 96 | 93 | 17.6 | -0.17 |
| A | A3 | Blood Culture  Bacterial/Yeast | Day 5 | Yes | No | 64 | 51 | 32.5 | -0.4 |
| A | A4 | Blood Culture  Bacterial/Yeast | Day 12 | Yes | No | 32 | 30 | 11.1 | -0.18 |
| B | B | Blood Culture  Bacterial/Yeast | Day 6 | Yes | No | 64 | 42 | 14.34 | -1.49 |
| C | C | Blood Culture  Bacterial/Yeast | Day1 | No | No | 30 | N/A | N/A | N/A |

Plasma ammonia (NH3); *Peak of trough plasma ammonia levels obtained after initial plasma ammonia level within 48 hours from time culture was obtained;  ^#^ Time interval between initial plasma ammonia level and either subsequent peak or trough ammonia level.


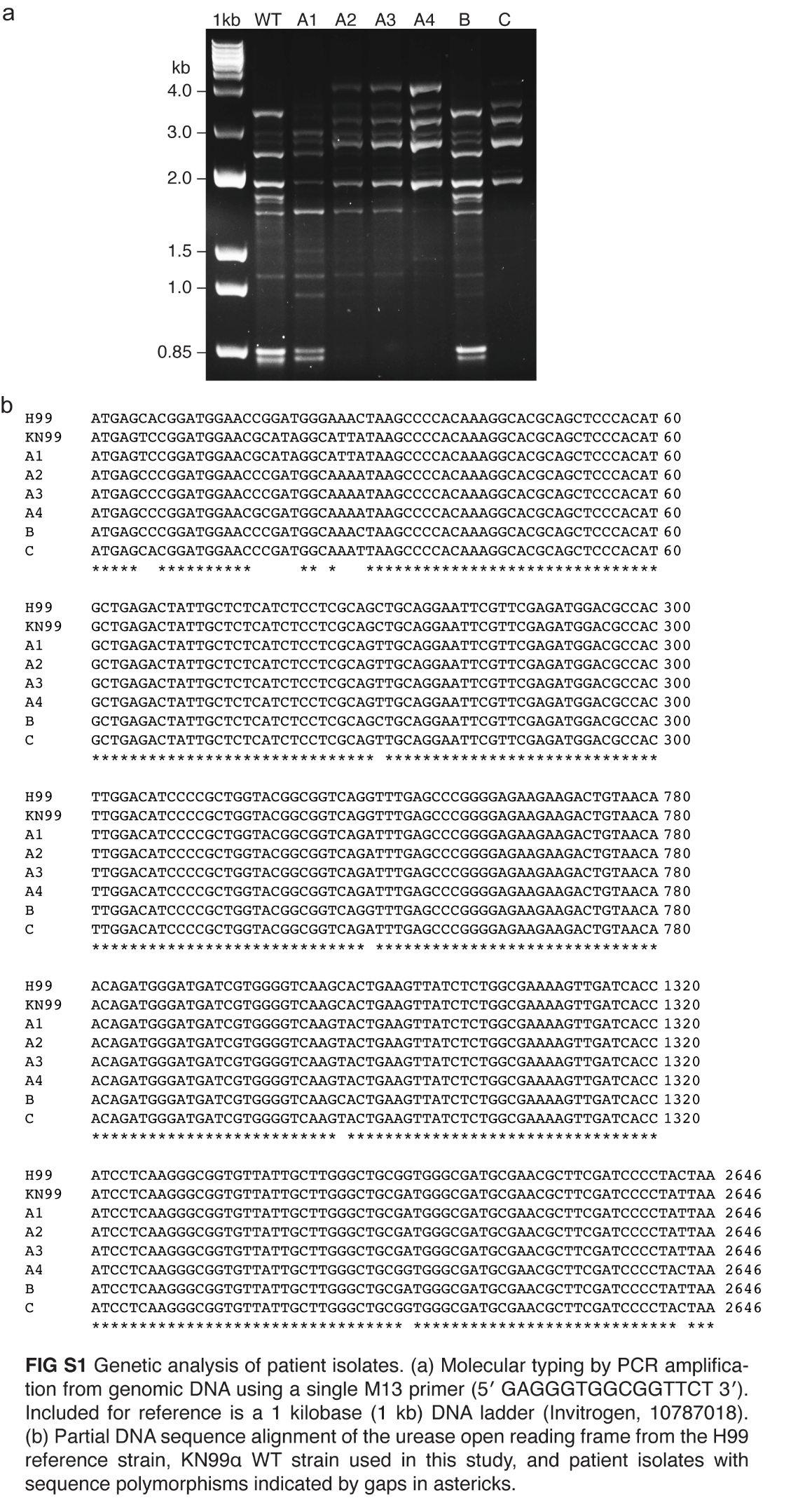


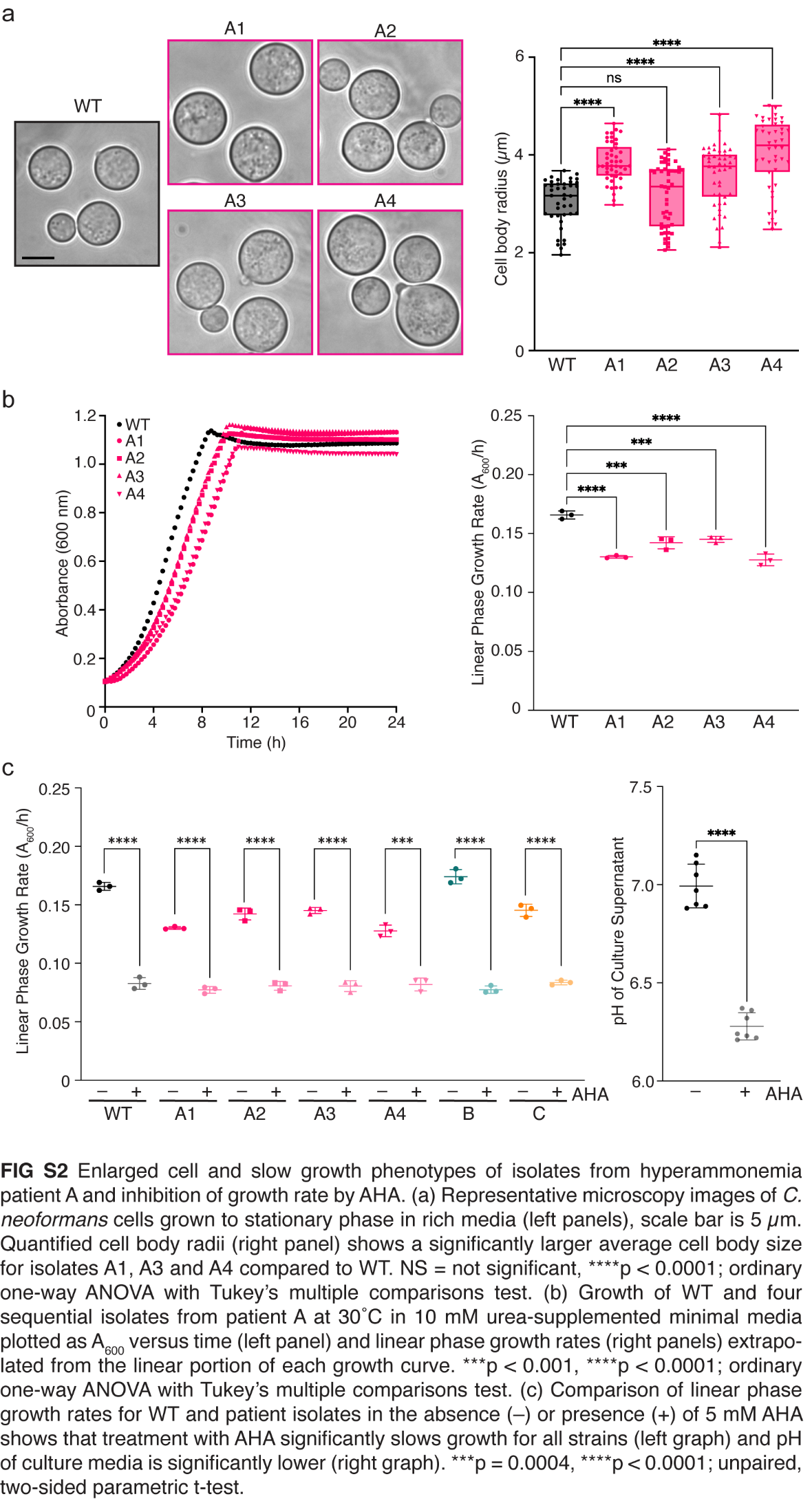


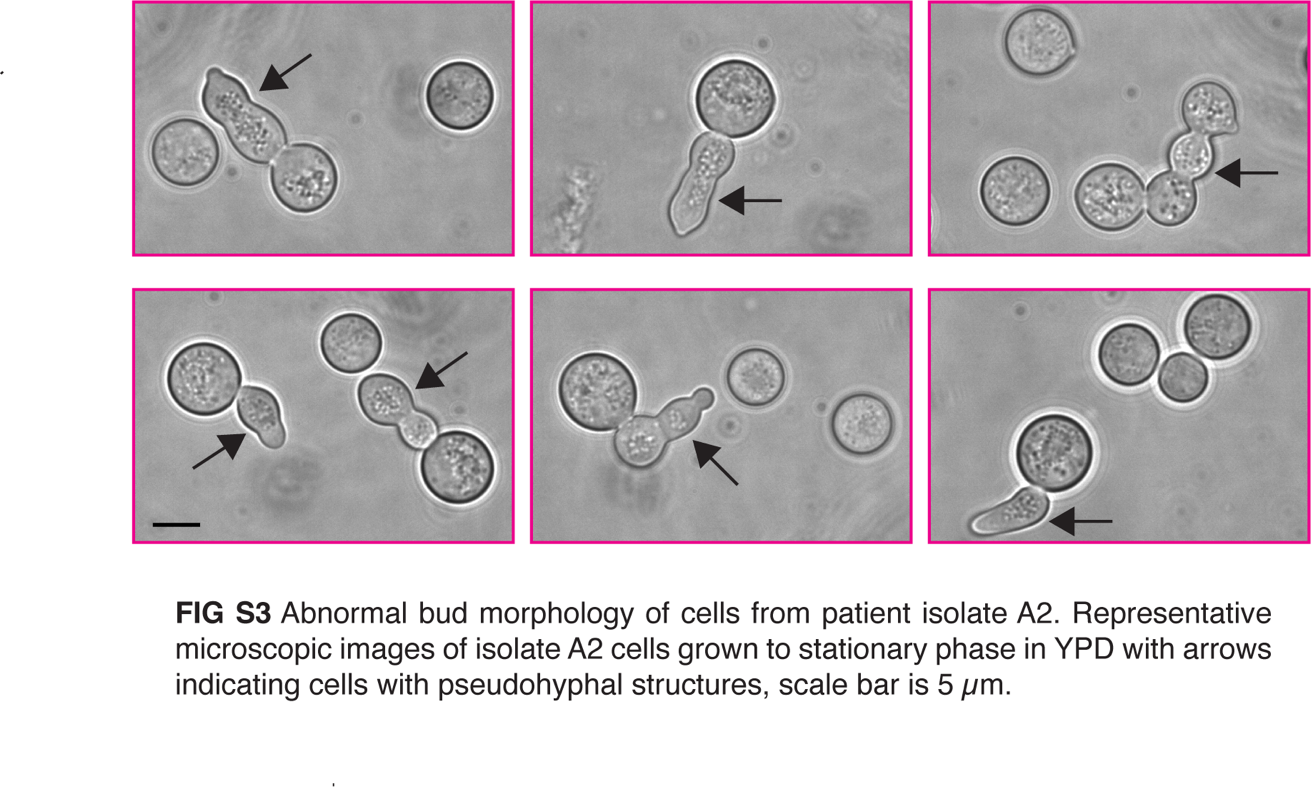


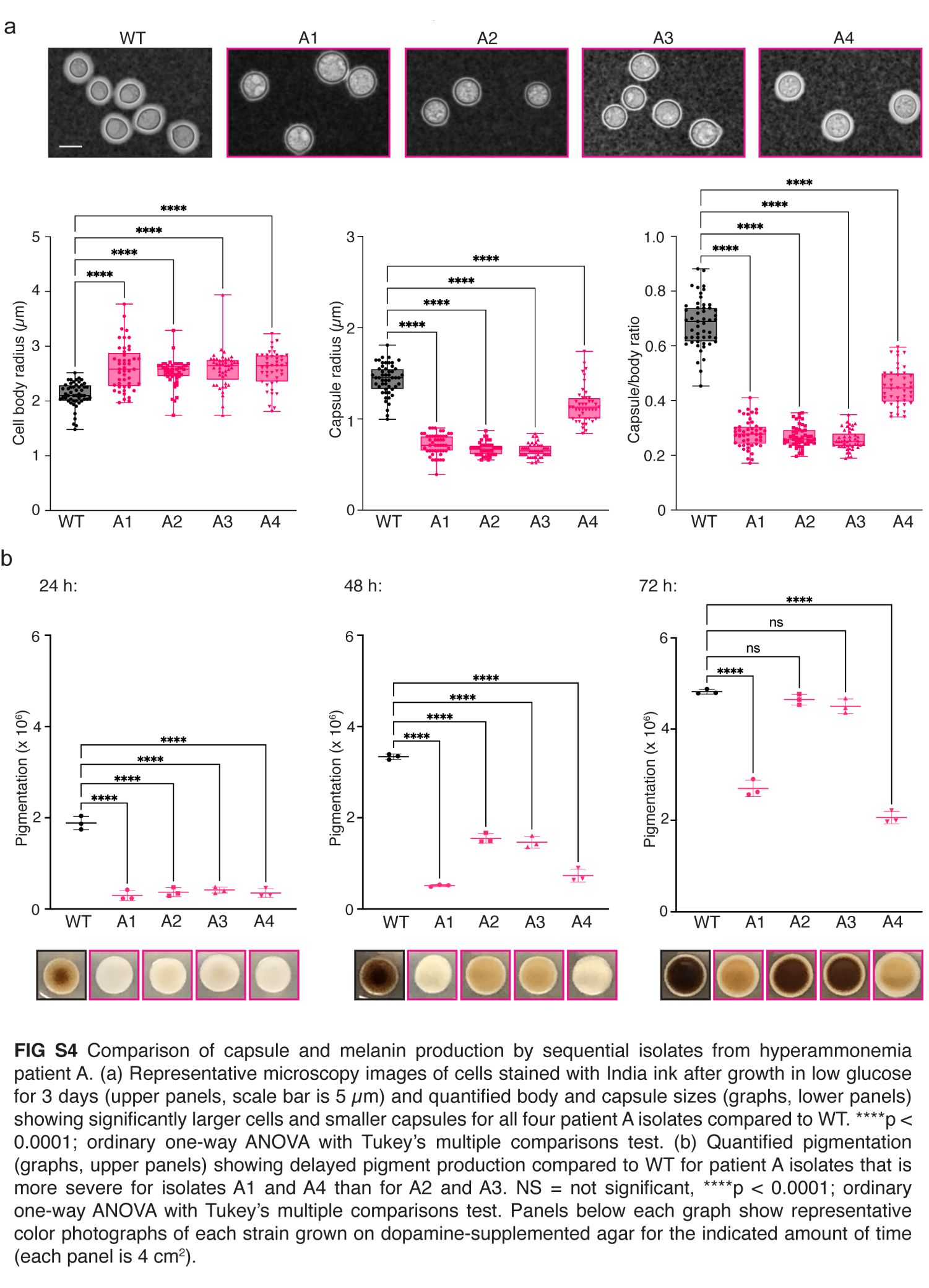
Figure S5: Flow chart of inclusion process for retrospective cohort study of plasma ammonia levels in 29 patients with *Cryptococcal* infections.

Emory data warehouse was queried using the following search parameters between 1/1/2014 to 9/30/2022:

1. All patients with positive *Cryptococcus neoformans* or *Gatii* blood and/or CSF culture

**and / or**

1. Positive *Cryptococca*l antigen in blood and/or CSF

**and**

1. Plasma ammonia level (>53 µmol/L) drawn during the same admission

**n= 35**

6 patients excluded due to ammonia levels drawn more than 7 days from a positive cryptococcal culture.

Clinical Study:

**n=29** included.

(28 with *C. neoformans* and 1 with *C. Gattii*)

Emory ICMC Biorepository queried for cryptococcal strains from 29 patients

*Cryptococcal* strains from 26 patients were not available at Emory ICMC Biorepository

Laboratory Study:

*Cryptococcal* strains from 3 patients were recovered from Emory ICMC biorepository:

Patient A (4 isolates from different dates)

Patient B ( 1 isolate)

Patient C ( 1 isolate)

Figure S6: Scatter plot with a linear fitted line showing the relationship between admission Blood Urea Nitrogen and peak NH3, Cryptammonia study, Emory data warehouse, 2013-2022 (N=28)


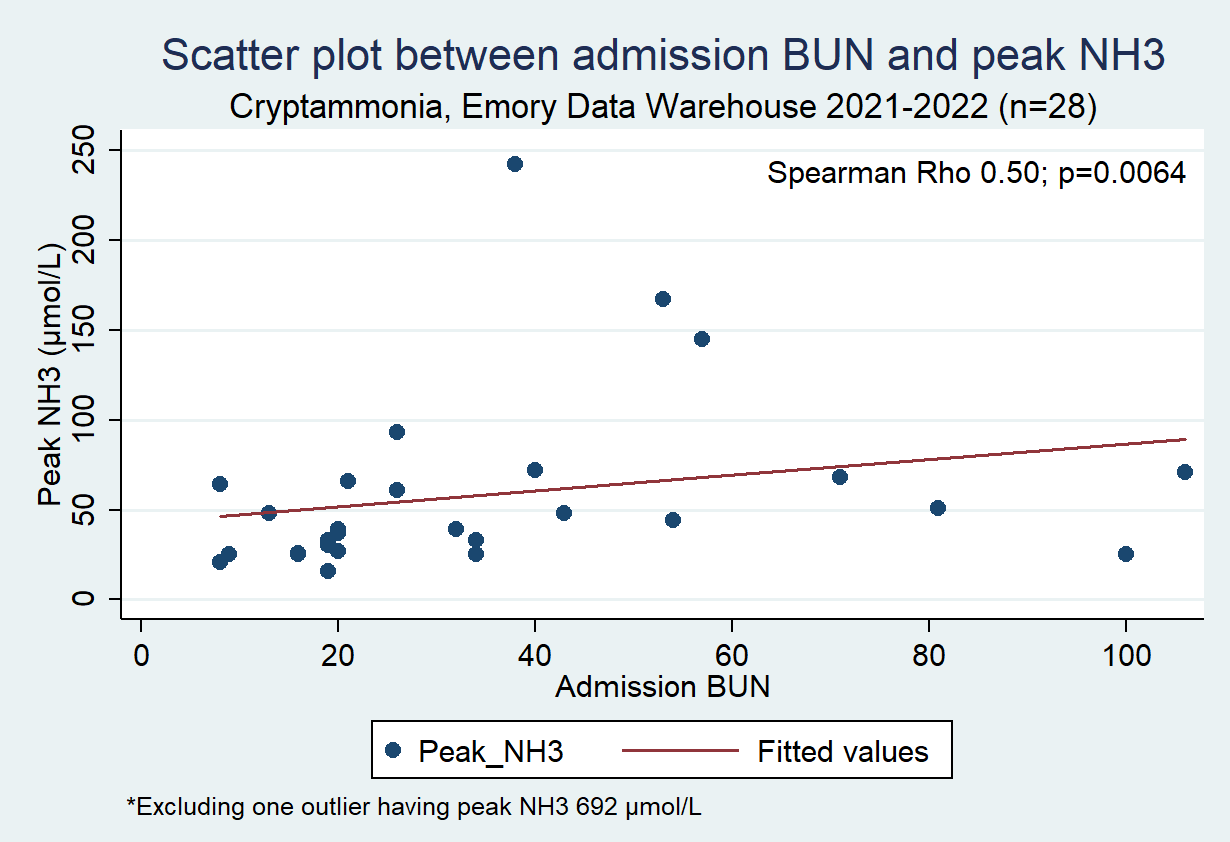

Supplement: Supplemental material — Table S1; Fig. S1 to S6. [file spectrum.03902-23-s0001.docx]
